# Genome Digging: Insight into the Mitochondrial Genome of *Homo*

**DOI:** 10.1371/journal.pone.0014278

**Published:** 2010-12-09

**Authors:** Igor V. Ovchinnikov, Olga I. Kholina

**Affiliations:** 1 Department of Biology, University of North Dakota, Grand Forks, North Dakota, United States of America; 2 Forensic Science Program, University of North Dakota, Grand Forks, North Dakota, United States of America; 3 New York, New York, United States of America; American Museum of Natural History, United States of America

## Abstract

**Background:**

A fraction of the Neanderthal mitochondrial genome sequence has a similarity with a 5,839-bp nuclear DNA sequence of mitochondrial origin (numt) on the human chromosome 1. This fact has never been interpreted. Although this phenomenon may be attributed to contamination and mosaic assembly of Neanderthal mtDNA from short sequencing reads, we explain the mysterious similarity by integration of this numt (mtAncestor-1) into the nuclear genome of the common ancestor of Neanderthals and modern humans not long before their reproductive split.

**Principal Findings:**

Exploiting bioinformatics, we uncovered an additional numt (mtAncestor-2) with a high similarity to the Neanderthal mtDNA and indicated that both numts represent almost identical replicas of the mtDNA sequences ancestral to the mitochondrial genomes of Neanderthals and modern humans. In the proteins, encoded by mtDNA, the majority of amino acids distinguishing chimpanzees from humans and Neanderthals were acquired by the ancestral hominins. The overall rate of nonsynonymous evolution in Neanderthal mitochondrial protein-coding genes is not higher than in other lineages. The model incorporating the ancestral hominin mtDNA sequences estimates the average divergence age of the mtDNAs of Neanderthals and modern humans to be 450,000–485,000 years. The mtAncestor-1 and mtAncestor-2 sequences were incorporated into the nuclear genome approximately 620,000 years and 2,885,000 years ago, respectively.

**Conclusions:**

This study provides the first insight into the evolution of the mitochondrial DNA in hominins ancestral to Neanderthals and humans. We hypothesize that mtAncestor-1 and mtAncestor-2 are likely to be molecular fossils of the mtDNAs of *Homo heidelbergensis* and a stem *Homo* lineage. The d_N_/d_S_ dynamics suggests that the effective population size of extinct hominins was low. However, the hominin lineage ancestral to humans, Neanderthals and *H. heidelbergensis*, had a larger effective population size and possessed genetic diversity comparable with those of chimpanzee and gorilla.

## Introduction

Hominin evolution is a complex process. It is symbolized by a bushy tree whose numerous branches represent morphological lineages with evolutionary relations that are not completely resolved. Genetics provides a set of powerful tools that can be used to clarify these connections. One approach is a comparison of genomes from closely related extant species: modern human (*Homo sapiens*) and common chimpanzee (*Pan troglodytes*). Unfortunately, comparison of the modern human and chimpanzee genomes cannot elucidate human evolution due to the tens of millions of independent mutation events in the both genomes that have accumulated throughout 6–8 million years of separate evolutionary trajectories. Explanation of the genetic bases of human evolution must thus emerge from the discovery of genetic variation in other hominid lineages. However, this task is challenging as endogenous DNA must be recovered from extinct hominin samples.

One of the ways to understand past genetic variation is a comparative analysis of genetic loci from human populations in order to make conclusions based on the age of different alleles. Such methods give some insight into the past but by no means unravel genetic variation that existed in other hominin lineages. It was proposed that genetic variants of the *MAPT* locus and the gene microcephalin [1], [2] were introgressed into the nuclear genome of *H. sapiens* during periods of coexistence between modern humans and archaic *Homo* lineages. However, these conclusions were not supported by the Neanderthal genome sequencing [3].

Another strategy is a resurrection of vanished genes by statistical inference of ancestral sequences at internal nodes on the phylogenetic tree [4]. This approach was once called paleogenomics [5], although now it is applied more broadly, including the DNA sequencing of ancient genomes [3], [6]. Several extinct amino acid and nucleotide sequences were reconstructed by this method, including those of the functional archosaur rhodopsin [7], the ancient vertebrate Hox1 gene [8], and the ancestral primate mitochondrial DNA (mtDNA) [9]. The advantages of this method include the possibility of functional analysis of extinct genes and experimental evaluation of evolutionary hypotheses. However, incorrect assumptions, stochastic errors and experimental artifacts may lead to misleading deductions [10].

The methodology of ancient DNA uses a direct approach to studying DNA from extinct organisms. As soon as the first ancient DNA was published [11], the key question emerged whether it is possible to use this technology for discovery of genetic differences among hominins, or at least between modern humans and the last extinct hominin group, the Neanderthals. The first discovery and independent confirmation of Neanderthal mtDNA [12], [13] have demonstrated the potential of such analysis. A decade of Neanderthal genetic study has resulted in the publication of the first mitochondrial genome sequence retrieved from several Neanderthal specimens from Vindija Cave [14]. The subsequent analysis yielded the additional five Neanderthal mitochondrial genome sequences with very high similarity to each other [15]. All of the Neanderthal mitochondrial genes demonstrated the absence of statistically significant evidence of selection. This result seemed a little disappointing to evolutionary biologists due to the absence of strong signs of adaptation to climate or diet in the mitochondrial genes. But it made it plausible to assume that neutral mutation processes under genetic drift played a major role in the accumulation of the genetic changes which distinguish the Neanderthal and modern human mtDNAs.

Understanding evolutionary changes in the mtDNAs of Neanderthals and modern humans may potentially come from their comparison with the mitochondrial genomes of their common ancestors. Unfortunately, the direct retrieval of mtDNA sequences from specimens representing other extinct *Homo* lineages remains elusive despite progress in the next-generation sequencing technologies.

However, to overcome the problem of recovering DNA from samples over 100,000 years old, a genetic approach exists that allows the reconstruction of the sequences of the mitochondrial genome ancestral to modern human and Neanderthal mtDNAs. This uses available nuclear genomes of modern humans. Our genomic landscape probably represents a small fraction of the genomic diversity that existed in ancestral African lineages of the genus *Homo*. Novel genetic alterations have been also added during independent evolution following the unprecedented dispersal of a small population of ancient *H. sapiens* that existed in East Africa 200,000 years ago. In such a mosaic genome there are DNA sequences that were inserted into the nuclear genome after the reproductive separation of the hominin and chimpanzee lineages. Among them numts, or nuclear DNA sequences of mitochondrial origin, represent replicas of vanished mtDNA molecules. In the human nuclear genome, the substantial majority of numts are neutrally evolving “mtDNA fossils”, in which the mean mutation rate is approximately ten times lower than the mean mutation rate in the mitochondrial genome [16].

Many of the numt insertions in the human genome are considered to be human-specific because they have integrated into the nuclear genome after the genetic divergence of hominins and chimpanzees. Today, these numts are unique to modern humans. However, a fraction of these insertions first emerged in other *Homo* lineages that subsequently died out, and before the appearance of morphological forms of *H. sapiens*. Due to our ignorance of the sequences of their mitochondrial counterparts from extinct hominin lineages, the precise time of their integration into the nuclear genome and their taxonomic association remains uncertain.

The retrieval of the first complete sequence of the Neanderthal mtDNA created a novel state which allows us to determine the time of transfer of mitochondrial DNA sequences into the nuclear genome, and to make conclusions about the mitochondrial gene sequences in the hominin lineages ancestral to *H. sapiens* and *H. neanderthalensis*. This approach is based on the comparison of unique human numt sequences with the Neanderthal mitochondrial genome, along with the mitochondrial genomes of modern humans and chimpanzees. Green et al. [14] reported a mysterious similarity between a 5,839-bp numt on the human chromosome 1, and 522 of 8341 mtDNA sequences from the 454 generated libraries obtained from the Vindija Neanderthal specimens. Remarkably, these Neanderthal mtDNA sequences have greater similarity to the human nuclear genome sequence than to human mtDNAs. There is a chance that this phenomenon is attributable to a problem of contamination and mosaic assembly of the Neanderthal mtDNA from short sequencing reads. However, this odd similarity may be also explained if we assume that the numt in question was inserted into the nuclear genome before *H. heidelbergensis* gave rise to the Neanderthal lineage [17]. In that case, this numt would represent a replica of the mtDNA sequence of *H. heidelbergensis*. We decided to explore this issue from an evolutionary viewpoint and to uncover additional numt sequences in the human nuclear genome that show higher similarity to Neanderthal mtDNA than modern human mtDNA.

## Results

### Collection of Numt Sequences

In order to find numts that were integrated into the nuclear genome in the hominin lineages ancestral to Neanderthals and modern humans, several criteria have been considered. First, such numts should be human-specific, with absence of their orthologous pairs in the chimpanzee genome. Second, numts should be represented in all modern continental groups, with sequences demonstrating a high level of conservation in different populations. Next, the numt sequences in the human nuclear genome should have a higher identity with the Neanderthal mitochondrial genome than with the mtDNA sequences of modern humans. Finally, positions of the numt sequences on phylogenetic trees should make evolutionary sense. These sequences will make up a separate branch, basal to both the Neanderthal and modern human sequences. This *Homo* clade would be distantly separated on phylogenetic trees from the group of chimpanzee and bonobo sequences.

To identify numts with such characteristics, a BLAT search was used in the Ensembl NCBI36 assembly of the human genome, with the Neanderthal mitochondrial genome as a query. We extracted a group of 1305 sequences. To find numt sequences with higher similarity to the Neanderthal mtDNA, the revised Cambridge reference (rCRS) mitochondrial genome [18] and an African mitochondrial genome belonging to the L0d lineage with the high level of divergence [19] were also utilized as queries. The BLAT searches revealed 1256 and 1251 sequences, respectively. The numt sequences that were ≥1000 bp long and had ≥90% identity were selected as the possible counterparts of the mtDNA sequences ancestral to the Neanderthal and modern human mtDNAs (Table 1).

**Table 1:** Numts selected by the BLAT search.

	Query			Chromosome			Neanderthal mtDNA		rCRS mtDNA		African mtDNA	
	Start	End	Length	Name	Start	End	Score	%ID	Score	%ID	Score	%ID
Numt-1	3909	9749	5842	1	554327	560166	28602	98.6	28584	98.55	28502	98.36
Numt-2	10264	15482	5219	5	134286898	134292116	23910	94.1	23904	94.06	23913	94.1
Numt-3	9768	14498	4740	5	99410222	99414952	19729	89.01	19803	89.08	- -	
Numt-4	953	2693	1742	5	79981597	79983333	8082	95.52	8075	95.46	8075	95.46
Numt-5	1289	2968	1680	11	10486010	10487689	7714	94.17	9223	94.15	9229	94.2
Numt-6	1392	2714	1324	3	97818722	97820044	6151	95.47	6151	95.47	6145	95.39

### Phylogenetic Analysis

**Figure 1:**
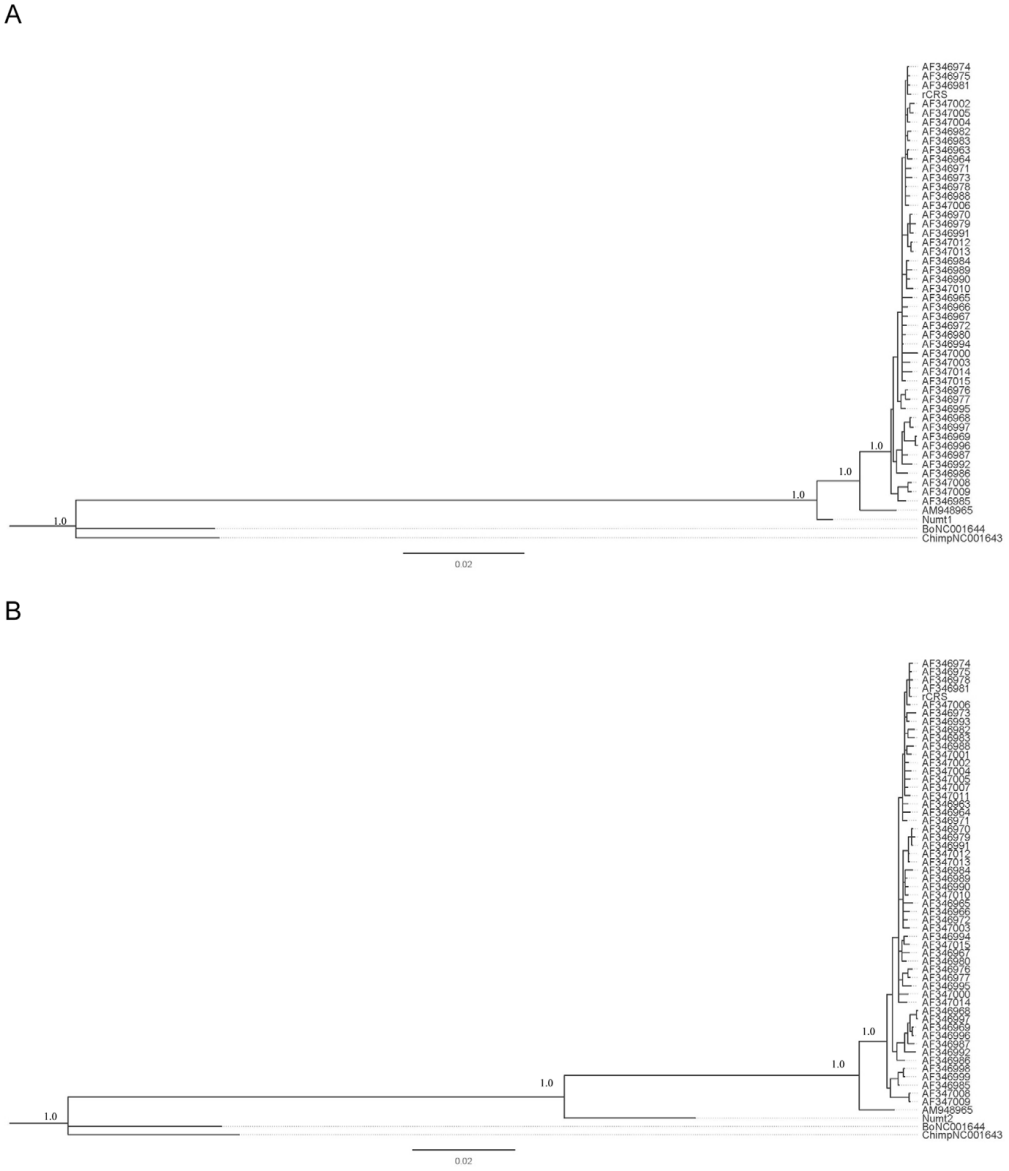
Bayesian inference phylogenetic trees. (A) Phylogenetic relationship of Numt-1 and the mtDNAs of 54 modern humans, one Neanderthal, one chimpanzee and one bonobo. (B) Phylogenetic relationship of Numt-2 and the mtDNAs of 54 modern humans, one Neanderthal, one chimpanzee and one bonobo. The numbers refer to the posterior probability values.

We conducted a phylogenetic analysis that included Numts 1–6 (Table 1) and the corresponding mtDNA sequences of one Neanderthal, 54 human mtDNAs, one chimpanzee and one bonobo. For consistency, we used the same set of human and chimpanzee mtDNAs as Green et al. [14]. The phylogenetic analysis indicated that only two of them, Numt-1 and Numt-2, cluster with the sequences of modern humans and Neanderthals. The similar topology of phylogenetic trees was demonstrated for both numts by neighbor-joining, maximum parsimony, and Bayesian methods. In these trees the modern human, Neanderthal, Numt-1 and Numt-2 sequences formed a macroclade that we called the macroclade *Homo*. Within the macroclade *Homo*, all modern human sequences were combined into a monophyletic group and the Neanderthal mtDNA sequence formed a distinct branch basal to modern humans. Both Numt-1 and Numt-2 appeared as outgroups to the Neanderthal and modern human taxa. The chimpanzee and bonobo sequences established another macroclade, the macroclade *Pan* (Figure 1).

We named Numt-1 mtAncestor-1 and Numt-2 mtAncestor-2, and these names will be used further in the text. The phylogenetic trees clearly show that the time of integration of each of the mitochondrial ancestor loci into the nuclear genome was different. MtAncestor-2 was transferred into the nuclear genome much earlier than mtAncestor-1. Thus, they were probably integrated into the nuclear genomes of different hominin species.

The phylogenetic analysis of other numts indicated that they do not cluster with the macroclade *Homo*. This result rejected them as potential replicas of the mitochondrial genome sequences of the common ancestor of Neanderthals and modern humans. In fact, these data complicated our attempt to reconstruct a complete mitochondrial genome ancestral to the mtDNAs of modern humans and Neanderthals. Nevertheless, they allowed us to investigate evolutionary processes in mitochondrial genes of hominins ancestral to *H. neanderthalensis* and *H. sapiens*.

### Characteristics of mtAncestor-1 and mtAncestor-2

mtAncestor-1 is a numt located on the human chromosome 1p36.33 (the NCBI36 position 554,327–560,166) whose sequence is aligned with the rCRS between positions 3914 and 9754. This numt contains the entire sequences of the genes encoding *tRNA-Ile*, *tRNA-Gln*, *tRNA-Met*, *ND2*, *tRNA-Trp*, *tRNA-Ala*, *tRNA-Asn*, *tRNA-Cys*, *tRNA-Tyr*, *COX1*, *tRNA-Ser*, *tRNA-Asp*, *COX2*, *tRNA-Lys*, *ATP8*, *ATP6*, and the incomplete sections of the *ND1* and *COX3* genes. mtAncestor-2 is a numt inserted into the human chromosome 5q31.1 (the NCBI36 position 134,286,898–134,292,116) with the sequence aligned with the rCRS between positions 10269 and 15487. This numt contains the entire sequences of the genes encoding *tRNA-Arg*, *ND4L*, *ND4*, *tRNA-His*, *tRNA-Ser*, *tRNA-Leu*, *ND5*, *ND6*, *tRNA-Glu*, and the incomplete sections of the *ND3* and *CYTB* genes. The nuclear genome of our ancestors had acquired these numts after the separation of hominin and chimpanzee lineages confirming the conclusion made earlier [20], [21], [22].

Before performing any manipulations with mtAncestor-1 and mtAncestor-2, we needed to demonstrate that their mitochondrial counterparts do not contain termination codons in inappropriate positions, as well as insertions and deletions which change the open reading frames of the mitochondrial genes. The mtAncestor-2 sequence contains the termination codons TAA in the amino acid position 35 in the *ND5* gene and in the amino acid position 108 in the *ND3* gene. In our analysis, we changed codon 35 in *ND5* to TAT and codon 108 in *ND3* to CAA as they are in the modern human and chimpanzee sequences. We also excluded codon 204 in the *COX2* gene from the analysis because the mtAncestor-1 sequence contains a deletion of AC in this codon.

### MtAncestor-1 and mtAncestor-2 are Conserved in Modern Humans

Today, several complete human genomes provide a rich virtual DNA sequencing material, although the analysis of some genome sequences is still challenging due to a low level of accuracy and internet portals without convenient bioinformatics tools. The mtAncestor-1 and mtAncestor-2 sequences from the human reference NCBI36 genome are completely identical to the corresponding numt sequences in both Watson's and Venter's genomes [23], [24]. The 100 Genome Project will soon provide the genome variation in multiple human populations [25]. To date, the 1000 genomes browser provides high-coverage sequencing data and alignments for one mother-father-child family of the European ancestry from Utah, U.S.A. and the other family trio of the Yoruba ancestry from Ibadan, Nigeria. The deep sequencing failed to detect any variation in the mtAncestor-1 sequence in the members of European and African families and in the mtAncestor-2 sequence in the European family. The mtAncestor-2 sequence indicates a variation in two nucleotide positions in the African family. In this family, one site demonstrates heterozygosity in both parents and the other site is heterozygous in each family member. We were unable to detect de novo mutations in these numts in both families.

The African and non-African human populations demonstrate substantial genetic diversity with the highest fraction of novel variants in Africa [25]. However, tens of thousands of years of the evolution of *H. sapiens* and the differentiation of the African and European populations that occurred on different continents have not led to accumulation of point mutations in mtAncestor-1 and mtAncestor-2, confirming a high level of conservation of these sequences after their integration into the nuclear genome.

### The Time of the Most Recent Common Ancestor

In order to understand the time of integration of the mtAncestor-1 and mtAncestor-2 sequences to the nuclear genome, we explored an approach based on a Bayesian Markov chain Monte Carlo algorithm for estimating species divergence times [26]. The program baseml was employed to analyze a molecular clock assumption. Due to different phylogenetic positions of the mtAncestor-1 and mtAncestor-2 sequences, we analyzed heterogeneity in mutation rates in two groups of DNA sequences. The first group of the sequences with the evolutionary recent divergence time is composed of 10 modern human mtDNAs, one Neanderthal mtDNA and mtAncestor-1. The likelihood ratio test failed to reject the null hypothesis that the mutation rate is homogeneous among all branches in the phylogeny (LR = 4.14 and p = 0.902). The addition of the mtAncestor-1 sequences made the statistical support of the molecular clock in this group of DNA sequences even stronger than it was observed by Green et al., 2008 [14]. The second group consisted of the single sequences belonging to different hominin lineages including modern humans, Neanderthals, an unknown hominin from Denisova cave [27] and mtAncestor-2. We added the Denisova mtDNA sequence taking into account its phylogenetic position. The comparison of our phylogentic tree (Figure 1, B) and the phylogenetic tree with the Denisova mtDNA sequence [27] allowed to conclude that the divergence of the Denisova sequence is two times older than the divergence of Neanderthal - modern human mtDNAs but approximately three times younger than the divergence of the mtAncestor-2 and modern human mtDNAs. We found that none of the analyzed sequences demonstrate significant heterogeneity in mutation rates (LR = 3.66 and p = 0.160). The absence of such heterogeneity permitted the molecular clock assumption to be applied to date the time of transfer of these numts into the nuclear genome.

To calculate the divergence date between the Neanderthal and modern human mtDNAs, Green et al. used a split time between humans and chimpanzees of 6 to 8 million years ago, based on the fossil record data [14]. This split time seemed to corroborate the molecular divergence of humans and chimpanzee at 5–7 million years [28] and the divergence age between the human and chimpanzee mitochondrial genomes at 6 million years [29].

The program mcmctree was used to estimate the time of the most recent common ancestor (TMRCA) of the mtAncestor-1 and mtAncestor-2 sequences and the mtDNAs of Neanderthal and modern humans under two scenarios, with the divergence date between modern humans and chimpanzees of 6–8 and 5–6 million years ago as based on fossil records and molecular data, respectively. For every scenario, we incorporated either no information about the divergence time between the human and Neanderthal mtDNAs, or a divergence date between these lineages of 520,000–800,000 years ago [14]. In total, four different models were analyzed and their results are shown in Table 2. We suggest that model 3 is the most plausible because it estimates the TMRCAs from the molecular divergence age of human and chimpanzee and without the preliminary divergence date of the modern human and Neanderthal mtDNAs.

**Table 2:** The time of the most recent common ancestor of mtAncestor-1 and mtAncestor-2 and the mtDNAs of Neanderthal and modern humans.

Models and their parameters			mtAncestor-1		
	T_div_ chimp & humans	T_div_ Neand & humans	TMRCA	95% credibility interval	Estimated T_div_ Neand & humans
1	6–8 MYA	na	785,000	550,000–1,070,000	570,000 (400,000–780,000)
2	6–8 MYA	500–800 KYA	815,000	630,000–1,050,000	585,000 (500,000–760,000)
3	5–6 MYA	na	620,000	440,000–820,000	450,000 (320,000–600,000)
4	5–6 MYA	500–800 KYA	710,000	590,000–870,000	535,000 (490,000–630,000)

Models and their parameters			mtAncestor-2		
Models	T_div_ chimp & humans	T_div_ Neand & humans	TMRCA	95% credibility interval	Estimated T_div_ Neand & humans
1	6–8 MYA	na	3,680,000	2,880,000–4,580,000	620,000 (450,000–830,000)
2	6–8 MYA	500–800 KYA	3,690,000	2,980,000–4,470, 000	575,000 (490,000–740,000)
3	5–6 MYA	na	2,885,000	2,350,000–3,480,000	485,000 (360,000–640,000)
4	5–6 MYA	500–800 KYA	3,030,000	2,560,000–3,540,000	525,000 (480,000–620,000)

T_div_, the divergence time.

According to model 3, the TMRCA of the mtAncestor-1 sequence and the mtDNA of Neanderthal and modern humans was estimated to be 630,000 years with a 95% confidence interval of 440,000–820,000 years. The divergence age of mtAncestor-2 is much lengthy - approximately 2,885,000 years with a 95% confidence interval of 2,350,000–3,480,000 years.

Using the same model, the TMRCA of Neanderthal and modern human mtDNA was estimated to be 450,000 years with a 95% confidence interval of 320,000–600,000 years for the first set of sequences and 485,000 years with a 95% confidence interval of 360,000–640,000 years for the second set of sequences. These TMRCAs precede a reproductive split between the Neanderthals and modern human lineages which approximately occurred 370,000–400,000 years ago [30], [31]. Taking into account the precision of the mtDNA molecular clock in the estimation of human origins and migrations, we conclude that model 3 provides more evolutionarily reliable TMRCA readings than the divergence age calculated by Green et al. [14].

### Evolutionary Forces Shaping the Mitochondrial Protein-Coding Genes

In order to reveal the evolutionary forces changing genetic variation in the mitochondrial protein-coding genes in Neanderthals, modern human and their common ancestors, we applied the program codeml which uses a likelihood approach to estimate nonsynonymous (d_N_) and synonymous (d_S_) substitution rates for branches in the phylogeny [32]. This approach can detect purifying (d_N_/d_S_<1) or positive (d_N_/d_S_ >1) selection acting on particular lineages. The mtDNA sequences of rCRS, Neanderthal, chimpanzee, bonobo, gorilla, orangutan, and baboon were aligned along with mtAncestor-1 and mtAncestor-2. The d_N_/d_S_ branch specific variations calculated from the average nucleotide frequencies at the three codon positions are shown in Table S1. For both sets of sequences, the background free d_N_/d_S_ ratios are lower than 1 and the branch specific d_N_/d_S_ ratios significantly vary from branch to branch (P<0.002 and P<0.005 χ^2^ test). Assuming Bonferroni's correction, only the common ancestral branch for the modern human, Neanderthal and mtAncestor-1 lineages indicates the significantly lower d_N_/d_S_ ratio than the background d_N_/d_S._ The lower d_N_/d_S_ ratio is likely caused by a large effective population size for a common ancestor of the human-Neanderthal-mtAncestor-1 mtDNA lineages which results in more efficient purifying selection.

Using the McDonald-Kreitman test, we attempted to determine a possible role of natural selection in the evolution of the protein-coding genes by a comparison of synonymous and nonsynonymous variation within a group of modern human mtDNAs, and between this group and the ancestral mtDNA sequences (Table 3). Except for the *CYTB* gene, the pattern of nucleotide changes in all other genes corresponds to the neutral model. The incomplete section (65%) of the *CYTB* gene shows statistically significant excess of nonsynonymous polymorphisms within modern humans. The largest value of the neutrality index also confirms increased amino acid variation in *CYTB* within modern humans. Interestingly, parsimonious nonsynonymous substitutions in *CYTB* are shared with the sole Neanderthal sequence which possesses two additional nonsynonymous substitutions. It appears that Neanderthals might also have had a significant excess of nonsynonymous polymorphisms in *CYTB* within their lineage.

**Table 3:** Synonymous and nonsynonymous substitutions in protein-coding genes within human mtDNA and between human and ancestral mtDNA lineages.

	Synonymous substitutions		Nonsynonymous substitutions		McDonald – Kreitman test	
Gene	Divergence between lineages	Human specific polymorphisms	Divergence between lineages	Human specific polymorphisms	P-value, Fisher's exact test	Neutrality index
mtAncestor-1 gene set						
*ND1* *	0	7	0	2	-	-
*ND2*	12	26	2	12	0.3	2.769
*COX1*	15	36	0	8	0.1	-
*COX2*	52	19	5	3	0.68	1.642
*ATP8*	5	6	0	1	1	-
*ATP6*	4	21	2	8	1	0.762
*COX3* *	7	19	1	4	1	1.474
mtAncestor-2 gene set						
*ND3* *	9	5	1	2	0.54	3.6
*ND4L*	9	9	3	1	0.59	0.333
*ND4*	40	43	6	12	0.3	1.86
*ND5*	78	51	30	23	0.74	1.173
*ND6*	16	20	5	5	1	0.8
*CYTB* *	41	17	7	11	**0.0024**	3.79

*Incompleted gene sequences.

Significant P-value is indicated in bold.

### Amino Acid Changes in the Mitochondrial Proteins

First, we tried to determine whether amino acid substitutions in the mitochondrial proteins which distinguish the human-Neanderthal lineage from chimpanzees were acquired uniquely in the former, or not. Alignment of the mitochondrial protein sequences indicated that the majority of these amino acid replacements (93 of 132) are not the unique acquisition of modern humans and Neanderthals but first emerged in the ancestral hominin lineages. The only exceptions are *COX2*, *ND5*, and *CYTB*, which intensively accumulated new amino acids in modern humans (*COX2*) or in modern humans and Neanderthals together (*ND5*, *CYTB*).

The second task was to identify unique amino acid substitutions in the ancestral mitochondrial genes in the comparison to 2704 modern humans [33], Neanderthal and chimpanzee mtDNA sequences. MtAncestor-1 has only one unique amino acid substitution in codon 191 in *ND2*. The homologous section of the Neanderthal mtDNA has two private amino acids in codons of 346 in *ND2* and codon 154 in *ATP6*. MtAncestor-2 has unique amino acid substitutions in codons 11 and 70 in *ND4L*, codons 55, 113, 317, 365, 420 in *ND4*, codons 8, 44, 56, 377, 480, 537 in *ND5*, codons 38, 73, 168 in *ND6*, and codons 153, 238 in the available part of *CYTB*. In this section of the mitochondrial genome, the Neanderthal sequence indicates two private amino acid substitutions in codons 489 and 603 in *ND5*.

It is important to understand whether unique amino acid substitutions in mtAncestor-1 and mtAncestor-2 are caused by mutation processes in mtDNA before the transpositions of these loci into the nuclear genome, or in the numt sequences themselves. Two criteria were used to identify unique mutations possibly acquired by these sequences after their insertion into the nuclear genome: (i) such unique mutations are unknown in mtDNA of other species and (ii) they have functional effects on encoded mitochondrial proteins.

According to the PolyPhen analysis, the majority of distinct amino acid substitutions are benign. However, amino acids in codon 56 (tyrosine) in *ND5*, codons 11 (glutamic acid) and 70 (lysine) in *ND4L*, and codon 73 (valine) in *ND6* might possibly have functional effects and change characteristics of encoded proteins. The PolyPhen program uses both structural-based and evolutionary-based approaches to predict potential effect of SNPs [34]. Of four amino acid substitutions with possible functional effects, a glutamic acid in *ND4L*'s codon 11 and a valine in *ND6*'s codon 73 are unknown in other species. Thus, we concluded that nonsynonymous nucleotide substitutions in these codons are likely caused by random mutational process after integration of mtAncestor-2 into the nuclear genome and excluded these mutations from our analysis.

### Assessment of DNA Damage in mtAncestor-1 and mtAncestor-2

We cannot exclude that some of nucleotide substitutions in mtAncestor-1 and mtAncestor-2 were due to random mutations that occurred after integration of these loci into the nuclear genome. The maximum mutation rate for noncoding, nonrepetitive genomic regions in the human genome has been determined as 0.99×10^−9^ substitutions per nucleotide site per year [35] and in such sequences approximately one mutation gets fixed every 200,000 years. Using visual inspection we found two nonsense mutations in codon 35 in *ND5* and codon 108 in *ND3* in mtAncestor-2 and one frameshift mutation in codon 204 in *COX2* in mtAncestor-1. Because these changes are highly deleterious in their mitochondrial counterpart and cannot persist in the mitochondrial genome, they must have been gained in the nuclear genome. Using the evolutionary based approach in PolyPhen, we also proposed that two nonsynonymous substitutions in codon 11 in *ND4L* and in codon 73 in *ND6* have been most likely acquired after integration of the mtAncestor-2 sequence into the nuclear genome.

We applied several lines of evidence to demonstrate that mtAncestor-1 and mtAncestor-2 are almost precise replicas of the mtDNA frozen since the moment of integration into the nuclear genome. The protein-coding genes in the mammalian mtDNA evolve under selective constraints and reveal codon position bias with the mean ratio of substitutions at 1^st^, 2^nd^, and 3^rd^ codon positions equal to 2∶1∶5 [36]. In contrast, after their integration into the nuclear genome, the functional sections of mtDNA become noncoding numts and begin evolving in the absence of selective constraints. Novel mutation in newborn numts will arise according to the neutral pattern of nucleotide substitutions hitting 1^st^, 2^nd^, and 3^rd^ positions of each triplet with equal probability.

Effective separation of mitochondrial and nuclear nucleotide substitutions is an intricate process due to the absence of apparent criteria. However, in some insect species with gigantic genomes and the highest reported rates of horizontal transfer between mitochondrial and nuclear genomes, unique substitutions in numts were counted as nuclear with the mean substitution ratio 1∶1∶1 at different codon positions [37], [38]. Although the evolutionary dynamics of numts in hominins is different, we investigated the patterns of private substitutions in mitochondrial genes of the functional counterparts of mtAncestor-1 and mtAncestor-2 which are not presented in the mtDNAs of 54 modern humans and one Neanderthal. Across all mitochondrial genes, the patterns of such substitutions are not significantly different from 2∶1∶5 (Table 4).

**Table 4:** Private nucleotide substitutions in the functional mitochondrial counterparts of mtAncestor-1 and mtAncestor-2.

	Position in codon					
Gene	1st	2d	3d	P-value	Transitions	Transversions
*ND1* *	-	-	-	-	-	-
*ND2*	1	2	7	0.287	10	0
*COX1*	1	0	9	-	8	1
*COX2*	0	0	7	-	7	0
*ATP8*	0	0	2	-	2	0
*ATP6*	2	0	3	-	3	2
*COX3* *	0	0	6	-	6	0
*ND3* *	2	0	5	0.535	7	0
*ND4L*	1	1	9	0.386	9	2
*ND4*	8	3	33	0.285	40	4
*ND5*	23	11	67	0.697	91	10
*ND6*	5	2	14	0.815	18	3
*CYTB* *	8	2	31	0.206	37	4

*Incompleted gene sequences.

Examination of the ratio of transitions (ts) to transversions (tv) causing these private substitutions in mtAncestor-1 and mtAncestor-2 is the next test allowing us to confirm that the pattern of nucleotide substitutions arose in the mitochondrial genome. In human nuclear DNA, transitions occur approximately twice as frequently as transversions [39]. In contrast, the ts/tv ratio is greater in human mtDNA, and may reach up to 15∶1 [40]. All mitochondrial genes except *ATP6* demonstrate a strong bias in favor of transitions in mtAncestor-1 and mtAncestor-2 (Table 4). These results confirm that the pattern of mutations in mtAncestor-1 and mtAncestor-2 arose in the mitochondria. The relatively low ts/tv ratio in *ATP6* may be plausibly explained by either stochastic variation of transitions and transversions among only five sites or the variable transition rate across mitochondrial genes.

As soon as mtDNA sequences transfer into the nuclear genome, they might have undergone elevated mutation rate in CpG dinucleotides caused by de novo cytosine methylation. In such sites, cytosine methylated nucleotides are potential targets of spontaneous deamination resulting in accidental CpG → TpG/CpA transition. We tested for the presence of DNA changes in CG doublets for nucleotide substitutions that led to unique amino acids in mtAncestor-1 and mtAncestor-2. Such changes were not found. This also confirms that the number of changes acquired after integration of mtAncestor-1 and mtAncestor-2 into the nuclear genome was very low.

Mitochondrial disease mutations were not expected in the mtAncestor-1 and mtAncestor-2 sequences because they are relatively rarely found in population studies of modern humans and have potentially low individual survival. For these reasons, mutations in mtAncestor-1 and mtAncestor-2 causing mitochondrial diseases are best interpreted as mutations that arose in the nucleus. The human mtDNA sequence corresponding to mtAncestor-1 may potentially carry 78 pathogenic single nucleotide mutations in protein-coding genes and 62 in RNA-coding genes [41], [42]. Of these 140 pathogenic mutations, none were found in mtAncestor-1. The human mtDNA sequence corresponding to mtAncestor-2 may prospectively bear 63 pathogenic single nucleotide mutations in protein-coding genes and 19 in RNA-coding genes [41], [42]. Of these 82 mutations, mtAncestor-2 has 10398G in *ND3*, which is associated with a reduced risk of Parkinson disease [43] and can be rejected as a disease mutation. This test confirms that the chance of spontaneous nuclear changes in these loci is very low indeed.

All lines of evidence considered above allow us to conclude that mtAncestor-1 and mtAncestor-2 represent almost identical replicas of the mtDNA sequences ancestral to the mitochondrial genomes of Neanderthal and modern humans.

## Discussion

### Taxonomy of mtAncestor-1 and mtAncestor-2

In order to attribute mtAncestor-1 and mtAncestor-2 to hominin lineages ancestral to Neanderthals and modern humans, we juxtaposed the divergence ages between different groups of DNA sequences with the evolution of *Homo* in the Pleistocene. Taking into account the African origin of modern humans and the presence of mtAncestor-1 and mtAncestor-2 in all continental populations of modern humans, we can definitely conclude that the incorporation of these numts into the nuclear genome occurred in Africa. These numts are also detectable in the first draft of the Neanderthal genome [3], although their sequences represented in this genome have a low accuracy in many parts.

The divergence time between the mtAncestor-1 sequence and the mtDNAs of Neanderthals and modern humans was calculated to be 620,000 years with a 95% confidence interval of 440,000–820,000 years. It points out that a transposition event occurred in an African Middle Pleistocene lineage ancestral to *H. sapiens* and Neanderthals and some representatives of this lineage possessed mtDNA sequences similar to mtAncestor-1. Although the human evolution in the Middle Pleistocene is controversial, the possible scenario assumes that this lineage might have belonged to *H. heidelbergensis* or to a closely related African lineage [44], [45]. Our results fit with the models of the late divergence and the intermediate divergence of Neanderthal from their African root group but do not support the early divergence hypothesis [46]. Thus, the mtAncestor-1 sequence provides the first insight into the mitochondrial DNA of an African branch of *H. heidelbergensis* [17].

The divergence age of mtAncestor-2 and the mitochondrial DNA sequences of the Neanderthal and modern humans was estimated to be 2,885,000 years with a 95% confidence interval of 2,350,000–3,480,000 years. According to the coalescent theory, the time of incorporation of an mtDNA fragment into the nuclear genome lags behind the TMRCA of a numt sequence and the sampled mtDNAs [47]. Close scrutiny of this divergence time and the chronology of the hominin taxa [48] make it improbable that mtAncestor-2 first appeared in the *Australopithecus* or *Paranthropus* lineages. While it is likely that transfer occurred in transitional forms such as *H. habilis* or *H. rudolfensis*, it is more plausible that it occurred in a stem population that led to *H. ergaster*. The oldest reportedly *Homo* fossils found in East Africa (Olduvai Gorge, Koobi Fora) dated to 2.3–2.5 million years ago might have potentially had mtDNA sequences congruent with mtAncestor-2.

### Ancient Demographic History and Variation in the dN/dS Ratio

The demographic history of extinct hominins is unknown. The codeml analysis with the incorporation of mitochondrial DNA sequences ancestral to the Neanderthal and modern human mtDNAs demonstrates that the d_N_/d_S_ ratios differ insignificantly among extinct and extant hominin lineages (Table S1). This variation may reflect the changes in the effective population size in different hominins as well as selection constraints in the mitochondrial protein-coding genes. We could not confirm that the d_N_/d_S_ ratio is significantly higher in the mitochondrial genes in Neanderthals than in modern humans [14].

The population structure of the Pleistocene hominins probably consisted of partially isolated subpopulations [49]. This model assumes that Neanderthals and modern humans evolved from different subpopulations of their common ancestor. The d_N_/d_S_ ratio indicates that the effective population size of their common ancestor (the human – Neanderthal branch) tends to be larger than either Neanderthals or modern humans.

The lineage ancestral to modern humans, Neanderthal and mtAncestor-1 is the only branch with significantly lower d_N_/d_S_ ratio than the background d_N_/d_S_ ratio. If our suggestion that mtAncestor-1 was a portion of the mitochondrial genome of *H. heidelbergensis* is correct, this ancestral lineage plausibly corresponds to *H. erectus* [50]. According to our observations, *H. erectus* as thus conceived had a larger effective population size and harbored greater genetic variation than Neanderthals and modern humans. The effective population size of *H. erectus* in this sense might have been closer to the effective population sizes of chimpanzee and gorilla. In contrast, the stem population that led to the radiation of the representatives of the genus *Homo* (the mtAncestor-2 branch) probably had a small effective population size (Table S1).

### The Evolution of Intracellular Functions and Physiological Differences

The functional roles of the majority of mtDNA mutations, as well as their evolutionary consequences, still remain unknown. Needless to say, the presence of DNA polymorphisms in mtAncestor-1 and mtAncesor-2 affecting intracellular processes may shed light on their role in the evolution of unique biomedical characteristics of modern humans. Astonishingly, the mtAncestor-1 and mtAncestor-2 sequences carry two closely linked polymorphisms at *ATP6* and *ND3* genes, 8701G and 10398G, which are involved in the regulation of mitochondrial matrix pH and intracellular calcium level. The guanine at the same positions is also found in the Neanderthal mitochondrial genome and in the most ancient mitochondrial DNA lineages among modern African people. In contrast, the ancestral 8701A/10398A allele is found in the chimpanzee and bonobo. Modern people demonstrate reversion to the ancestral allele in different populations belonging to all continental groups.

Cells with the 8701G/10398G mtDNA have higher mitochondrial matrix pH and possess higher activity of complex I in the electron transport chain than cells with the nonsynonymous substitutions 8701A and 10398A in their mtDNA [51]. The 10398G polymorphism is strongly associated with longevity [52] and exerts a protective effect against Parkinson's disease [43]. In contrast, the 10398A polymorphism is associated with vulnerability to complex diseases in modern humans including bipolar disorder [53], [54] and Alzheimer's disease in men [55]. The latter associations are most likely due to decreased activity of the mitochondrial electron transport chain [56]. Although we have not found any reported association of these SNPs with schizophrenia, oxidative phosphorylation and ATP synthesis are among the most significantly downregulated pathways in the prefrontal cortex of schizophrenia patients [57].

The evolution of hominins has been accompanied by tremendous changes in the size, complexity and relative proportions of the brain. The process of encephalization requires additional energy and more effective synthesis of ATP. This physiological process is coordinated by numerous genes, including the genes of mitochondrial genome that encode components of the mitochondrial electron transport chain. In the process of evolution of anthropoid primates the amino acid replacements in these genes led to more rapid transport of electrons along with the expansion of neocortex [58]. However, the connection between the evolution of the mitochondrial genome and the human brain has not been explored so far.

Our observation allows us to form a hypothesis about the involvement of the polymorphisms at ATP6 and ND3 in the process of encephalization in hominin lineages, and their contribution to the recent origin of disorders affecting cognitive, emotional and behavioral functions in modern humans. It is likely that the ancestral 8701A/10398A allele is a component of intricate physiological mechanisms maintaining energy metabolism sufficient for a brain of the size and complexity found in chimpanzees and their common ancestor with humans. However, this energy balance might have proved insufficient to sustain metabolic processes in larger brains within *Homo*.

We have determined that the 10398G polymorphism, and hence neurons with higher activity of the mitochondrial electron transport chain, might have first appeared in hominins with the mtAncestor-2 – like mtDNA sequences. The presence of 8701G/10398G in mtAncestor-1 and mtAncestor-2 suggests that acquisition of these linked polymorphisms occurred more than two million years ago. In that case, the substitution of 8701A/10398A for 8701G/10398G preceded brain enlargement in *H. habilis* [59] and led to better energetic balance for an enlarged brain of higher complexity. It is not unlikely that the persistence of the 8701G/10398G allele in different *Homo* lineages was one of the genetic factors which contributed to the process of encephalization.

The ancestral forms of *ATP6* and *ND3* might have independently emerged again in modern humans in the process of adaptating energetic balance to a decrease in average absolute brain size in *H. sapiens* [59]. Decreased activity of mitochondrial complex I in neurons associated with the ancestral allele probably added a novel risk for manifestation of complex cognitive disorders because the metabolic capabilities of the human brain had potentially reached their limits. That suggests the occurrence of a critical threshold in energetic balance of neurons [60]. If the mitochondrial genome has played an important role in the evolution of the human brain, our conclusion places an additional brick in the foundation of the theory that schizophrenia and bipolar psychosis are costs of the complexity of the human brain [61], [62]. We can hypothesize that some other cognitive conditions associated with the highly complex central nervous system are also human-specific, and arose recently during the evolution of archaic and modern humans.

## Materials and Methods

### Identification of Numts

The BLAT search was carried out to search numts in the Ensembl NCBI36 assembly of the human genome sequence (www.ensembl.org). The revised Cambridge reference (GenBank accession number J01415.2), Neanderthal (AM948965) and African (EF184591) mitochondrial genomes were used as queries.

### Phylogenetic Analysis

MtDNA sequences from 54 modern humans (J01415.2, AF346963 – AF347015), Neanderthal (AM948965), common chimpanzee (NC_001643.1) and bonobo (NC_001644.1) were aligned along mtAncestor-1 and mtAncestor-2 using Mega 4.1 [63]. The neighbor-joining (NJ) and maximum parsimony (MP) phylogenetic analyses were performed with Mega 4.1. The Bayesian inference of phylogeny was conducted with MrBayes 3.1.2 [64]. For the NJ analysis, the maximum composite likelihood model was used with a gamma distribution of 0.2 and 10,000 bootstraps. For the MP tree, default parameters with 100 bootstraps were utilized. For the Bayesian estimation of phylogeny, the GTR model was employed with the parameters described in Green et al. [14].

### Calculation of the Time of the Most Recent Common Ancestor

The program baseml of PAML 4.1 [65] and the likelihood ratio test were used to test a molecular clock in two groups of DNA sequences. The first group included 10 modern human mtDNAs, Neanderthal mtDNA and mtAncestor-1 sequences. The modern human mtDNAs were selected according to the criteria described in Green et al. [14]. The second group included one modern human mtDNA sequence which was selected randomly, Neanderthal mtDNA, Denisova mtDNA and mtAncestor-2 sequences. Because the likelihood ratio test failed to reject the molecular clock assumption for the two groups of sequences, the program mcmctree of PAML 4.1 [65] was used to estimate divergence times. The data set included the model HKY85 with the parameters identical those used in Green et al. [14].

### Detection of Selection Acting on Particular Lineages

The mtDNA sequences of rCRS, Neanderthal, common chimpanzee, bonobo, gorilla (NC_001645.1), orangutan (NC_001646.1), and baboon (NC_001992.1) were aligned along mtAncestor-1 and mtAncestor-2 using Mega 4.1. Codons with insertions and deletions and sequence sections with overlapping reading frames were deleted. The program codeml of PAML 4.1 [65] was used to estimate the d_N_/d_S_ branch specific variations under one-, free-, and two-ratio models calculated from the average nucleotide frequencies at the three codon positions (the model F3×4). The χ^2^ statistical test was performed to confirm that free-ratio model fits the data significantly better than the one-ratio model. Bonferroni's correction with the significance level 0.004 was used to test each two-ratio model.

### Detection of Selection Acting on Particular Genes

The McDonald-Kreitman test [66] and the estimation of neutrality index [67] were performed for each of the protein-coding genes in a group of 54 modern human and either of mtAncestor-1 and mtAncestor-2 with DnaSP 4.50.3 [68].

### Analysis of Amino Acid Changes

The analysis of functional effects of amino acid changes on encoded mitochondrial proteins was carried out by PolyPhen (http://genetics.bwh.harvard.edu/pph/references.html).

## Supporting Information

Table S1 Likelihood Ratio Test of the d_N_/d_S_ Branch Specific Variations.
(0.06 MB DOC)
