# Supplementary material for: Genome Digging: Insight into the Mitochondrial Genome of Homo
Source: PLoS One. 2010 Dec 9;5(12):e14278. doi: 10.1371/journal.pone.0014278 (PMC3000329; doi:10.1371/journal.pone.0014278)
Supplement: Table S1 — Likelihood Ratio Test of the dN/dS Branch Specific Variations. (0.06 MB DOC) [file pone.0014278.s001.doc]

**Table S1** Likelihood Ratio Test of the d_N_/d_S_ Branch Specific Variations.

| Model | 2Δl | df | P-value | | Background d_N_/d_S_ | Branch specific d_N_/d_S_ |
| --- | --- | --- | --- | --- | --- | --- |
| Sequences aligned along mtAncestor-1: | | | | | | |
| One ratio | na | na | na | 0.0443 | | na |
| Free-ratio, 12 branches | 30.08 | 11 | **<0.002** | na | | na |
| Two-ratio, Neanderthal branch | 6.16 | 1 | <0.02 | 0.0433 | | 0.1387 |
| Two-ratio, modern human branch | 3.52 | 1 | >0.05 | 0.0436 | | 0.107 |
| Two-ratio, mtAncestor-1 branch | 0.58 | 1 | >0.1 | 0.0445 | | 0.021 |
| Two-ratio, modern human - Neanderthal branch | 0.32 | 1 | >0.5 | 0.0441 | | 0.0617 |
| Two-ratio, modern human - Neanderthal - mtAncestor-1 branch | 10.6 | 1 | **<0.002** | 0.0476 | | 0.0201 |
| Two-ratio, chimpanzee branch | 0 | 1 | >0.99 | 0.0443 | | 0.0443 |
| Two-ratio, bonobo branch | 3.04 | 1 | >0.05 | 0.0431 | | 0.075 |
| Two-ratio, *Pan* branch | 1.1 | 1 | >0.1 | 0.0452 | | 0.0327 |
| Two-ratio, gorilla branch | 0.46 | 1 | 0.5 | 0.0451 | | 0.0389 |
| Two-ratio, orangutan branch | 6.74 | 1 | <0.01 | 0.0404 | | 0.058 |
| Two-ratio, baboon branch | 2.68 | 1 | >0.1 | 0.0469 | | 0.0376 |
| Sequences aligned along mtAncestor-2: | | | | | | |
| One ratio | na | na | na | 0.0612 | | na |
| Free-ratio, 12 branches | 26.78 | 11 | **<0.005** | na | | na |
| Two-ratio, Neanderthal branch | 1.46 | 1 | >0.1 | 0.0606 | | 0.1104 |
| Two-ratio, modern human branch | 5.38 | 1 | <0.025 | 0.0599 | | 0.168 |
| Two-ratio, mtAncestor-2 branch | 3 | 1 | >0.05 | 0.0595 | | 0.1021 |
| Two-ratio, modern human - Neanderthal branch | 0.02 | 1 | >0.5 | 0.061 | | 0.0634 |
| Two-ratio, modern human - Neanderthal - mtAncestor-2 branch | 2.66 | 1 | >0.1 | 0.0629 | | 0.0361 |
| Two-ratio, chimpanzee branch | 4.84 | 1 | <0.05 | 0.059 | | 0.109 |
| Two-ratio, bonobo branch | 5.5 | 1 | <0.02 | 0.0588 | | 0.1109 |
| Two-ratio, *Pan* branch | 3.48 | 1 | >0.05 | 0.0632 | | 0.036 |
| Two-ratio, gorilla branch | 1.88 | 1 | >0.1 | 0.0635 | | 0.0491 |
| Two-ratio, orangutan branch | 0.02 | 1 | >0.5 | 0.0613 | | 0.0607 |
| Two-ratio, baboon branch | 2.52 | 1 | >0.1 | 0.0641 | | 0.0514 |
| 2Δl, likelihood ratio statistics; 2Δl = 2(l_1_ – l_0_), where l_0_ is the log likelihood value under one-ratio model and l_1_ is the log likelihood value under either free-ratio or two-ratio model.  df, degrees of freedom.  Significant P-values are indicated in bold. | | | | | | |
